# Fertility Awareness Methods Are Not Modern Contraceptives: Defining Contraception to Reflect Our Priorities

**DOI:** 10.9745/GHSP-D-16-00044

**Published:** 2016-06-20

**Authors:** Kirsten Austad, Anita Chary, Alejandra Colom, Rodrigo Barillas, Danessa Luna, Cecilia Menjívar, Brent Metz, Amy Petrocy, Anne Ruch, Peter Rohloff

**Affiliations:** a Wuqu’ Kawoq | Maya Health Alliance, Santiago Sacatepéquez, Guatemala; b Boston Medical Center, Department of Family Medicine, Boston, MA, USA; c Population Council, Guatemala City, Guatemala; d Asociación Alas, Antigua, Guatemala; e Asociación Generando Equidad (ASOGEN), Chimaltenango, Guatemala; f University of Kansas, Department of Sociology, Lawrence, KS, USA; g University of Kansas, Center for Latin American and Caribbean Studies, Lawrence, KS, USA; h Friendship Bridge, Panajachel, Guatemala; i SewHope, Petén, Guatemala; j Brigham and Women’s Hospital, Division of Global Health Equities, Boston, MA, USA

## Abstract

A recent article in GHSP calls for classifying fertility awareness methods as “modern contraceptives” despite their inferiority. We believe in a rights-based approach, which considers the real-world conditions that many women face, including constrained sexual agency and low baseline reproductive health literacy. We must demonstrate true commitment to increasing access to the most effective and reliable contraceptive methods.

See related article by Malarcher.

## INTRODUCTION

Unintended pregnancy is both a global public health challenge and an important human rights issue.^1^ Worldwide 40% of pregnancies are unintended.^2^ These unintended pregnancies pose significant health risks to women because of the obstetrical risks of multiple births, short interpregnancy intervals, and unsafe abortions, as well as because they worsen poverty-related inequalities. Addressing this unmet need for family planning mandates a coordinated response of dedicated human resources, economic investment, and application of the best-available scientific evidence. Highly efficacious and safe methods of contraception including injectable and oral contraceptives, sterilization, and long-acting reversible contraceptives (LARCs), comprising implants and intrauterine devices (IUDs), are key to this effort.

Global data on the use of various forms of contraception are important for understanding rates of unplanned pregnancies, monitoring unmet contraceptive needs, and tracking user preferences. For this reason, the term “modern contraceptives” has been introduced as an umbrella term grouping together barrier methods, injectable and oral contraceptives, LARCs, and sterilization. While no precise consensus on the term “modern contraceptives” exists, one compelling definition claims they “are technological advances designed to overcome biology” that “enable couples to have sexual intercourse at any mutually desired time.”^3^

“Modern contraceptives” is an umbrella term describing barrier methods, injectables, oral contraceptives, implants, IUDs, and sterilization.

In the March 2016 issue of *Global Health: Science and Practice*, Malarcher et al.^4^ advocate, on behalf of the United States Agency for International Development (USAID), that fertility awareness methods (FAMs) should be included in the definition of modern contraceptives. We see this proposed change in terminology as a step in the wrong direction toward the goal of fulfilling every woman’s right to plan her family.

We see Malarcher and colleagues’ call for fertility awareness methods to be classified as modern contraceptives as a step in the wrong direction.

## DRAWBACK OF FAMS

The argument for FAMs as modern contraceptives hinges on the assertion that they are “effective at pregnancy prevention.” Malarcher et al. support this claim by citing similar typical-use effectiveness rates for the Standard Days Method and TwoDay Method as barrier methods, and for the Lactational Amenorrhea Method (LAM) as injectable and combined oral contraceptives. The authors, however, fail to highlight that FAMs are outperformed by LARCs with efficacy rates of 99.95% for the currently available implant, 99.8% for the levonorgestrel IUD, and 99.2% for the copper IUD, all within the first year of use.^5^ These differences become even more pronounced when extended over the decades of a woman’s reproductive lifetime, a time frame more relevant to patients.

Also missing from Malarcher et al.’s article is a discussion of the significant limitations of FAMs that limit their real-world utility. Both the Standard Days Method and TwoDay Method assume that a woman has the agency to say no to intercourse during her fertile period, a choice not available to the 10% to 50% of women who experience sexual violence and coercion worldwide.^6^ This limitation is of particular importance for adolescent girls, up to one-third of whom report that their first sexual experience was forced, according to the World Health Organization’s (WHO’s) “World Report on Violence and Health.”^7^

The Standard Days Method and TwoDay Method assume women have the agency to say no to sex during her fertile period.

Additionally, LAM is by definition only a temporary solution to be used in the immediate 6-month postpartum period. Six months is shorter than the medically recommended interpregnancy interval of about 3 years,^8^ and thus LAM must, at best, be conceived as only a bridge to a longer-term method. Moreover, LAM is ineffectual for the 20% to 81% of eligible women who will begin menstruating before 6 months postpartum, despite exclusive breastfeeding.^9^ Sudden return of menses during a period in which a woman anticipated she would have had reliable contraception leaves her vulnerable to an unplanned pregnancy.

The Lactational Amenorrhea Method is only a temporary solution to be used in the immediate 6-month postpartum period.

## TIERED-EFFICACY COUNSELING AND POLICY

Malarcher and colleagues also argue that redefining FAMs as modern contraceptives will facilitate increased investments in their introduction and provision. But in what way does that prioritization of resources serve the needs of vulnerable women? Ideally, family planning counseling should be non-directive. It should help a woman clarify her unique desires and preferences, and it should effectively provide her with the information necessary to make her informed personal choice. To this point, research shows that the *absolute effectiveness* of a method for pregnancy prevention is *the most important factor* cited by end users when choosing a method, even over other considerations such as side effects.^10^,^11^

Method effectiveness is the most important factor cited by end users when choosing a method.

An important aid in educating women on their choices is the evidence-based WHO tiered-efficacy graphic, which provides pictures of all contraceptive methods in 4 rows, beginning with the most effective types (LARCs and sterilization) in the top row and ending with the least effective methods (withdrawal and spermicides) in the bottom row. Despite such visual aids, communicating actual-use failure rates in a way women can easily understand can be difficult.^12^,^13^ Clearly communicating to women which forms of contraception are most effective and reducing barriers to their access must be public health priorities.

Categorizing FAMs as modern contraceptives, as Malarcher et al. seek to do, is counterproductive to these goals. Their proposed shift in terminology risks sending an incorrect message to women, medical providers, and policy makers that we should think of all contraceptive methods as equally effective under real-world conditions. For a useful analogy within another field of medicine, consider a patient with high cholesterol for which there are 2 treatments, one of which is 20 times more effective than the other at preventing deadly heart attack and stroke (roughly the same efficacy of LARCs vs. non-LARCs for pregnancy prevention^14^). In this situation, the patient is best served if the physician first draws attention to the most effective treatment, including a fair assessment of side effects and other drawbacks, and then continues on to the entire list of less efficacious options, none of which need be withheld if the patient’s values and goals are not met by the first-line treatment. We should approach undesired fertility with this same seriousness and with the same fair assessment of efficacy data because decisions about contraception can also be lifesaving. This is true for many of the 47,000 women who die each year seeking unsafe abortions for undesired pregnancies, which are largely preventable by existing, highly efficacious contraceptive methods.

The proposed shift in terminology for fertility awareness methods sends an incorrect message that we should think of all contraceptives as equally effective under real-world conditions.

In this discussion, it is important not to place the most effective contraceptive methods such as LARCs in opposition to other family planning methods. We do not mean to suggest that the capacity for provision of these most effective contraceptive methods should completely replace teaching on FAMs. However, all modern, non-FAM methods require intentional investments in infrastructure and human capital. Terminology that overestimates the real-world efficacy of FAMs may serve to undermine these necessary investments, such as securing supply chains for LARCs and increasing trained providers, which underlie the current crisis of access to family planning in many low- and middle- income countries. In the final analysis, this end may be best served by replacing the vocabulary of modern and traditional contraception with a classification system based solely on method effectiveness, such as that provided by the WHO tiered-efficacy chart. Vulnerable women around the world are best served both by individualized counseling and national or regional policies that prioritize the most effective methods of pregnancy prevention, as women themselves continue to request.^10^^,^^11^

The best solution may be to replace the vocabulary of modern vs. traditional contraception by classifying methods solely on their effectiveness.

## A RIGHTS-BASED APPROACH

Maintaining choice in decisions regarding contraception is fundamental—a point which Malarcher and colleagues do effectively point out—and one which is a basic issue of human rights.^15^ A woman may choose any method of contraception for reasons as varied as personal perception of side effects, cultural norms, religious beliefs, or prior negative experience with a method, all of which must be respected.

The core challenge is teaching counselors to remain sensitive to these factors while also clarifying misinformation about effective contraception. For example, studies have catalogued a multitude of factual misperceptions among end users about LARCs, such as beliefs that IUDs cause infertility or chronic pelvic pain or that they lead to reproductive malignancies.^16^ If we do not develop effective techniques for ensuring that women correctly understand the medical facts, then we are providing “free choice” in name only. Categorizing FAMs as modern contraceptive techniques, by overestimating their real-world effectiveness and the level of autonomy and agency that women can exercise when deploying them, only exacerbates this central issue.

## ON THE GROUND

The authors of this piece are united by our work for reproductive justice in Guatemala. A lower-middle-income country, Guatemala provides a case study of the systemic barriers that compromise women’s ability to plan their fertility. Guatemala has one of the highest total fertility rates in Latin America. According to a large-scale national survey, 56% of women do not want to have more children. One in 3 Guatemalan women has an unmet need for a modern contraceptive method (excluding FAMs), and this need is even more marked among indigenous women and adolescent girls.^17^

The utility of FAMs is greatly restricted in Guatemala, because many women have limited power to choose whether or not to engage in sexual intercourse. Sexual violence was a commonly used military weapon during the country’s civil war from 1960 to 1996 and remains unsettlingly prevalent today. Similarly, poor understanding of fertility means that FAMs often fail to meet the family planning needs of adolescents, who give birth to 1 in every 5 children born in Guatemala.^17^ The country’s Ministry of Education, tasked with offering sexual education in public schools since 2010, has delayed its implementation under pressure from religious authorities and prominent political figures. As a result, women have little access to formal public sexual health education necessary to help them make more informed reproductive choices. Many of our patients, clients, and beneficiaries report that sex and sexuality were taboo topics within their households when they grew up, and women’s baseline understanding of their menstrual and fertility cycles are low. Promoting FAMs within these contexts of constrained sexual agency and low baseline reproductive health literacy is an immediately dangerous strategy.

We strongly believe tiered-efficacy family planning efforts are part of the solution to these pressing human rights problems. How we talk about contraception reflects our priorities to women and to the global health community. Terminology used to classify methods should reflect women’s desires for highly efficacious contraception and should reflect the realities of women’s social positions and restricted agency in many settings. This end is best served by clearly stating that modern contraceptive methods and FAMs are *not* equal tools. We hope that USAID and other policy and funding agencies will work to expand women’s access to safe and effective contraceptive technology, in defense of the human right to reproductive freedom.

## Notes

**Peer Reviewed**
